# Creosote in Ophthalmia

**Published:** 1854-10

**Authors:** E. K. Rich

**Affiliations:** Magnolia, Ill’s.


ART. III—*Creosote in ophthalmia, by* E. K. Rich, *M. D.*

Messrs. Editors.—I have been making a trial of Creosote in the treatment of the Catarrhal Ophthalmia, which, by the way, has been quite prevalent in this section during the past spring and summer; not being satisfied in all cases with the nit. silver, especially those cases attended with high inflammatory action, and knowing the peculiar good effects of Creosote in inflammations about the mouth, I was induced to try it for the eyes, and since I have commenced using it, I have used *nothing else* in the shape of lotion. I have tried it of different strengths dissolved in rain water, and have found two drops Creosote to the ounce of water strong enough to commence with in acute cases, gradually increasing the strength up six drops to the ounce.

This solution I consider far superior to the nit. silver, as it does not cause the eye to smart except for a few seconds, which is immediately followed by a clearing of the sight—the patients usually expressing themselves in a minute or so, as seeing as well as ever. I have, in a few cases, had to use counter irritation, but in the great majority of cases, have cured without it.

My object in sending this to you is to call the attention of my medical brethren to the article, in order to have its virtues in this complaint more fully tested.

Magnolia, Ill's. Sept. 18, '54.

